# *MT3‐MMP* down‐regulation promotes tumorigenesis and correlates to poor prognosis in esophageal squamous cell carcinoma

**DOI:** 10.1002/cam4.790

**Published:** 2016-06-12

**Authors:** Zengfu Xue, Xiumin Wu, Xiong Chen, Qi Luo

**Affiliations:** ^1^ Xiamen Cancer HospitalThe First Affiliated Hospital of Xiamen UniversityXiamenFujianChina; ^2^ Department of PharmacyThe First Affiliated Hospital of Xiamen UniversityXiamenFujianChina; ^3^ Department of Medical OncologyThe Affiliated Dongfang Hospital of Xiamen UniversityFuzhouFujianChina

**Keywords:** Cell cycle, esophageal squamous cell carcinoma, immunohistochemistry, *MT3‐MMP*, prognosis

## Abstract

The membrane‐type matrix metalloproteinases (MT‐MMPs) play an important role in degrading the extracellular matrix (ECM) and facilitating protease‐dependent tumor progression and invasion. Here, we report that unlike *MT1‐MMP, MT3‐MMP* was down‐regulated in esophageal squamous cell carcinoma (ESCC) as detected by real‐time PCR (qPCR), Western blot analysis, and immunohistochemistry (IHC). Down‐regulation of *MT3‐MMP* was observed at protein level in 66.3% of ESCC specimens (by IHC, *n* = 86) for routine pathologic diagnosis, as well as at mRNA level in 63.3% of surgically resected ESCC tumors paired with surrounding nontumor tissues (by qPCR, *n* = 30). Notably, *MT3‐MMP* down‐regulation significantly correlated with lymph node metastasis and poor overall survival of patients with ESCC (median 5‐year survival = 50.69 vs. 30.77 months for patients with *MT3‐MMP*‐negative and ‐positive ESCC, respectively). Mechanistically, *MT3‐MMP* negatively regulated proliferation, colony formation, and migration of ESCC cells, in association with cell cycle arrest at G1, due to up‐regulation of p21^Cip1^ and p27^Kip1^. Together, as a tumor suppressor in ESCC, *MT3‐MMP* down‐regulation represents an unfavorable factor for prognosis of patients with ESCC.

## Introduction

Esophageal squamous cell carcinoma (ESCC) is one of the most common cancer types and a leading cause of death worldwide, with a much larger prevalence in Asia than in Western countries 1, 2. Although advances in surgery (e.g., excision with clear margins) have recently emerged in ESCC treatment, the 5‐year overall survival rate of ESCC patients remains very low (10–16%) 1, 2. The proteolytic enzyme matrix metalloproteinases (MMPs) are closely related to cancer metastasis. During disease progression to metastatic stage, MMPs are usually up‐regulated and/or activated to allow invasion of tumor cells through the basement membrane of esophageal epithelia, an event essential for tumor metastasis to distant organs 3. Among various members of the MMP family, membrane‐type 1 matrix metalloproteinase (*MT1‐MMP*) is known as a key enzyme, which often associates with the transition of tumor cells to an invasive phenotype 3, 4. However, unlike *MT1‐MMP* and *MT2‐MMP*, which promote tumor cell invasion, the role of *MT3‐MMP*, another membrane‐type matrix metalloproteinase, remains largely unclear in cancer 5.

MMPs represents a family of zinc‐dependent multidomain endopeptidases, which are responsible for degradation of virtually all structural components of extracellular matrix (ECM), as well as numerous bioactive molecules, thereby playing essential roles in a variety of physiological and pathological events, especially cancer metastasis 6, 7. *MT3‐MMP* or *MMP16*, as a membrane‐anchored matrix metalloproteinase, is a major mediator of pericellular matrix proteolysis involved in the remodeling of extracellular matrix, either directly through execution of proteolysis or indirectly by activating other enzymes 8. For example, *MT3‐MMP* can activate pro‐MMP‐2 9, while MMP‐2 is known to promote cancer cell invasiveness 10. It is also recognized that *MT3‐MMP* has stronger activities in degrading extracellular matrix than *MT1‐MMP*, partially due to activation of MMP‐2 11. Moreover, *MT3‐MMP* is critical for governing the transition of tumor cells to an invasive phenotype 8, 12, 13, 14, 15.

However, the initial notion that MMPs act as metastasis‐promoting enzymes has been challenged by most recent findings 16. Despite the established pro‐tumorigenic roles of certain MMPs (e.g., MMP2, MMP9, and *MT1‐MMP*), recent studies have demonstrated that some MMPs such as *MMP8* and *MMP11* might act against tumor growth and metastasis. For example, *MMP8*‐deficient mice, when challenged with carcinogens, display a markedly increased susceptibility to tumorigenesis, compared to wild‐type mice 17. Further, it has been shown that higher *MMP8* levels correlate to lower risk of distant metastasis, as well as better prognosis of patients with breast or oral cancer 16. Similar antitumor effects or dual (i.e., anti‐ and pro‐tumorigenic in a context‐specific manner) functions have also been found in the case of several other MMPs, including MMP11, MMP12, MMP19, and MMP26.

To date, the expression status and clinical role of *MT3‐MMP* in ESCC remains virtually unknown. Here, we report that *MT3‐MMP* was down‐regulated in ESCC tumor tissues, which correlates to high metastasis rate and poor survival; therefore, representing a favorable factor for prognosis of patients with ESCC. These findings were further validated mechanistically using ectopic overexpression and shRNA knockdown of *MT3‐MMP* in an in vitro model of human ESCC.

## Materials and Methods

### Cell lines

The human ESCC cell lines EC109 and EC9706 were obtained from the Chinese Academy of Medical Science (Beijing, China). Cells were cultured in RPMI‐1640 medium (GIBCO, Carlsbad, CA) containing 10% heat‐inactivated fetal calf serum (GIBCO, Carlsbad, CA) in a humidified incubator at 37°C in the presence of 5% CO_2_.

### Patients and primary tumor specimens

A total of 86 patients diagnosed as ESCC between July 2006 and October 2008 were enrolled into this study at the First Affiliated Hospital of Xiamen University and Xijing Hospital. Patient clinicopathologic characteristics are described in Table**** 1. The median age was 56 years (ranged from 32 to 73 years). Among them, 78 patients were followed up till the end of this study, that is, till September 2013, while eight patients were lost to follow‐up. Disease‐specific survival of patients was defined as a period of time from the date of diagnosis to the date of cancer‐related death or the end of the study. Primary tumor specimens for immunohistochemistry (IHC) analysis were obtained from patients undergoing routine procedures of pathologic diagnosis.

**Table 1:** Relation between MT3‐MMP expression and clinicopathologic characteristics in esophageal squamous cell carcinoma (ESCC) (*n* = 86)

Characteristics	No. of patients	MT3‐MMP positive,*n* (%)	*P* value
Age at diagnosis, year			0.647
≥60	58	21 (24.4)	
<60	28	8 (9.3)	
Sex			0.996
Male	64	21 (24.4)	
Female	22	8 (9.3)	
Tumor invasion			0.386
T1	5	2 (2.5)	
T2	12	3 (3.5)	
T3	65	23 (27.1)	
T4	3	0 (0)	
Differentiation			0.004
Well + moderate	68	28 (32.6)	
Poor	18	1 (1.2)	
Lymph node metastasis			0.039
N0	49	21 (24.4)	
N1	21	6 (7)	
N2	9	2 (2.3)	
N3	7	0 (0)	
Resected lymph node metastasis			0.017
Negative	57	29 (31.4)	
Positive	29	7 (8.1)	
Clinical stage			0.056
I	7	3 (3.5)	
II	46	20 (23.3)	
III	33	6 (7)	

Among 86 patients, thirty underwent esophagectomy. None of them had received preoperative chemotherapy. Fresh surgical specimens of primary ESCC tumor and surrounding nontumor (~5 cm away from the tumors) tissues were collected, stored in liquid nitrogen, and later used for qPCR and Western blot analyses. The pathologists evaluated each cancer specimen.

This study was approved by the Ethical Committee of the First Affiliated Hospital of Xiamen University. The informed consents were signed by all patients.

### Immunohistochemistry

Immunohistochemical analysis was performed as described before 18, using an ESCC Microarray Kit (Outdo Biotech, Shanghai, China) in primary tumors and their adjacent nontumor tissues of all 86 patients with ESCC. Anti‐*MT3‐MMP* antibody (ZA‐0266, 1:100, BA1280,Boster, Wuhan, China) was used as primary antibody, with which tissue sections were incubated overnight at 4°. Sections were incubated with anti‐mouse/goat IgG as negative controls. Two independent pathologists blindly evaluated each specimen. Immunostaining intensity was scored as 0–3 (0, negative; 1, weak; 2, moderate; and 3, strong). The percentage of *MT3‐MMP*‐positive cells (staining intensity score ≥1) was determined and then scored as weak (10–30%, +1), moderate (30–60%, +2), and strong (>60%, +3).

### Western blot analysis

Tumor and nontumor tissues obtained from the surgical specimens of 30 ESCC patients, as well as cells of the ESCC cell lines, were lysed by ultrasonication and then incubated with radio‐immunoprecipitation assay (RIPA) buffer. After loading the proteins (30 *μ*g) onto an 8% SDS‐PAGE, electrophoresis was performed at 20 mA for 60 min under denaturing conditions, and the proteins were then transferred to a nitrocellulose membrane. The membrane was incubated in 5% fat‐free milk for 1 h. 30 *μ*g of protein per condition was subjected to SDS‐PAGE and then electrotransferred onto nitrocellulose membrane. Anti‐*MT3‐MMP* antibody (ZA‐0266, 1:100, Boster) was used as primary antibody. Horseradish peroxidase (HRP)‐conjugated anti‐rabbit or anti‐goat antibodies (1:5000; Sigma‐Aldrich) were used as secondary antibody. Blots were visualized by an enhanced chemiluminescence (ECL) system (Amersham Pharmacia Biotech, Arlington Heights, IL). Each blot was repeated three times.

### Real‐time PCR (qPCR)

Total tissue RNA was isolated from 30 surgical specimens using the TRIzol reagent (Invitrogen) as recommended by the manufacturer, and DNase was used to block the contamination of genomic DNA. cDNA was synthesized from 2 *μ*g of total RNA per condition using a PrimeScript Reverse Transcription Reagent Kit and SYBR premix Ex Taq (Takara, Dalian, China). PCR amplification was then carried out as follows: initial denaturation at 95°C for 2 min, followed by 45 cycles of 95°C for 15 sec, 56°C for 20 sec, and 72°C for 15 sec. Reference for quantitation was human GAPDH as housekeeping gene. The primers include: *MT3‐MMP*, forward 5′‐TTCGTCGTGAGATGTTTGT‐3′, reverse 3′‐CCCGCCAGAAGTAAGTAA‐5′; GAPDH, forward 5′‐GCACCGTCAAGGCTGAGAAC‐3′, reverse 3′‐TGGTGAAACGCCAGTGGA‐5′.

### RNA interference, plasmids, and transfection

EC109 and EC9706 cells at 60–80% of confluence were transfected using Lipofectamine 2000 (Invitrogen, Carlsbad, CA), with either a *MT3‐MMP*‐pcDNA3.1 plasmid (kindly provided by Dr. Stephen J. Weiss, University of Michigan) in different concentrations and/or constructs encoding shRNA specifically targeting human *MT3‐MMP*‐1, *MT3‐MMP*‐2, or scrambled sequence as negative control. The following sequences were used for shRNA: negative control, 5′‐GCAAAGAAGGCCACTACTATA‐3′; *MT3‐MMP*‐1, 5′‐TAAGCCAATCACAGTCTGGAAATTCAAGAGATTTCCAGACTGTGATTGGCTTTTTTTTC‐3′; *MT3‐MMP*‐2, 5′‐TCGAGAAAAAAAAGCCAATCACAGTCTGGAAATCTCTTGAATTTCCAGACTGTGATTGGCTTA‐3′. The pLV‐3.7 lentiviral vector containing shRNA oligo was used to generate lentiviruses as per the manufacturer's instructions (Biosettia). Cells were harvested for protein and mRNA analyses 72 h after transfection. Alternatively, stable clones were selected with 600 *μ*g/mL G418.

### Cell cycle analysis

Cells were seeded onto 60‐mm‐well plates and incubated overnight in complete medium, followed by incubation for 48 h in serum‐free medium to synchronize cells, and then in complete medium to release from synchronized block. At 24 h of release, cells were harvested, washed with ice‐cold PBS, fixed with 70% ethanol, and then stained with 20 *μ*g/mL propidium iodide (PI) containing 200 *μ*g/mL RNase A (Sigma). Flow cytometry was then carried out to determine cell cycle distribution.

### Colony formation and wound healing assays

Colony formation assays were performed to measure the survival and proliferation of cells. Briefly, one thousand cells were seeded onto 6‐cm dishes and cultured for 2 weeks. After fixing with 70% ethanol and stained using Giemsa Stain, colonies were counted under a microscope. Alternatively, to further measure colony formation in anchorage‐independent condition, cells were plated in 0.3% soft agar over a base of 0.5% agar in complete medium. After culturing for 3 weeks, the number of colonies > 100 *μ*m was counted.

Wound healing assay was conducted to assess cell migration. Briefly, EC109 cells were grown to create a confluent monolayer and then scraped with a p200 pipet tip to create a scratch. Images for three randomly selected fields per condition were captured at the indicated intervals (0–72 h) and then analyzed quantitatively using a computing software.

### Statistical analysis

Values represent the means ± SD for at least three independent experiments performed in triplicate. Significance of differences between variables was determined using the chi‐square, Fisher's exact, and Wilcoxon rank‐sum tests. *P* < 0.05 was considered statistically significant. Kaplan–Meier survival analysis and multivariate analysis (Cox proportional hazards regression model) were used to analyze overall survival of patients. All data analyses were conducted using the SPSS 21.0 software package (Chicago, IL).

## Results

### *MT3‐MMP* is down‐regulated in primary ESCC tumors

**Figure 1:**
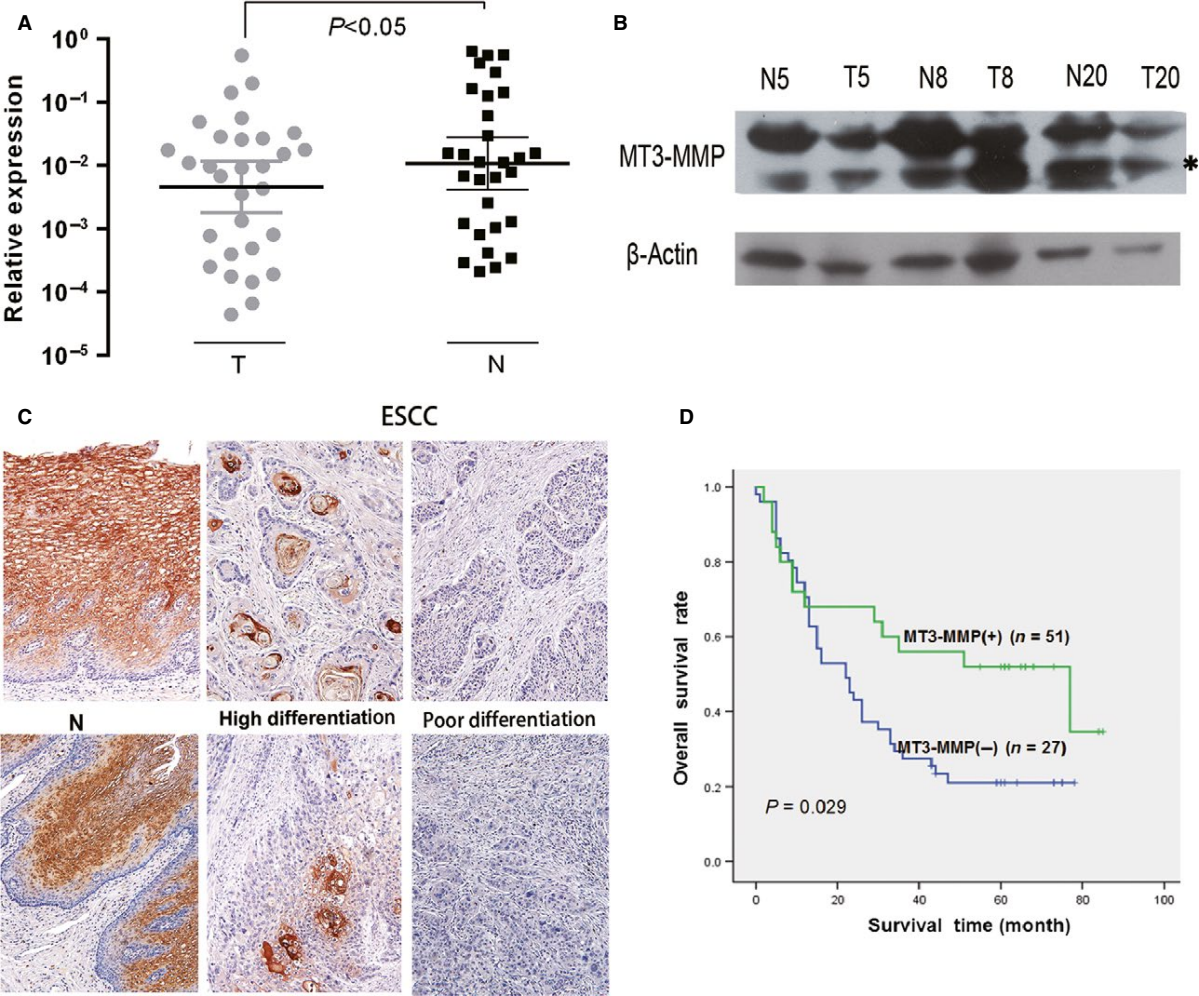
MT3‐MMP is down‐regulated in ESCC, correlating to poor patient survival. (A) Thirty paired primary specimens were obtained from surgical resection of ESCC tumor, after which qPCR was performed to relative mRNA levels of MT‐MMP in tumor (T) and nontumor (N) tissues (*P *<* *0.05, paired Student's *t*‐test). GAPDH was used as the reference for quantitation. (B) Alternatively, Western blot analysis was performed to monitor protein levels of MT3‐MMP in the indicated samples, in order to validate mRNA expression described in panel 1A. * indicates unknown band. (C) In 86 primary samples obtained for routine pathologic diagnosis, immunohistochemical staining (IHC) using anti‐MT3‐MMP antibody was performed to assess expression of MT3‐MMP. Representative images are shown for the adjacent nontumor tissue (left) and tumor tissues of well‐differentiated (middle) or poorly differentiated ESCC (right). Magnification, ×100. (D) In parallel, Kaplan–Meier analysis was performed to assess the effect of MT3‐MMP expression on overall survival (OS) rate of patients with ESCC. While eight patients were lost to follow‐up during the study, a total of 78 patients were eligible for analysis of OS. MT3‐MMP positive (+) expression was defined as score ≥1 (i.e., 1–3) for intensity of IHC staining, while score = 0 for MT3‐MMP negative (−). Green line, patients with MT3‐MMP (+) ESCC (median OS, 50.69 months); blue line, patients with MT3‐MMP (−) ESCC (median OS, 30.77 months. *P *=* *0.029 for comparison between MT3‐MMP (+) and (−) groups.

To examine the potential relationship between *MT3‐MMP* expression and disease progression of ESCC, qPCR was first performed to monitor mRNA expression of *MT3‐MMP* in ESCC tumors and their paired surrounding nontumor tissues of 30 surgical specimens. qPCR revealed that *MT3‐MMP* was down‐regulated in 19/30 (63.3%) of primary ESCC tumors, compared to their nontumor counterparts (*P* < 0.05, Fig.**** 1A). In some representative cases whose *MT3‐MMP* mRNA levels were markedly higher in nontumor than tumor tissues, Western blot analysis confirmed down‐regulation of *MT3‐MMP* at protein level in ESCC tumors (Fig.**** 1B). IHC revealed that *MT3‐MMP* was highly expressed in the membrane and cytoplasm of cells within nonkeratinized stratified squamous epithelium of nontumor esophageal tissues (left panel). However, *MT3‐MMP* positivity was largely seen in cells within the horny pearl in tumor tissues of moderately and well‐differentiated ESCC (middle panel, Fig.**** 1 **C**). In contrast, no or very low *MT3‐MMP* staining was detected in tumor tissues of poorly differentiated ESCC (right panel, Fig.**** 1 **C**).

### Down‐regulation of *MT3‐MMP* correlates to lymph node metastasis in ESCC patients

To assess the clinical significance of *MT3‐MMP* in disease progression of ESCC, *MT3‐MMP* expression was further examined by IHC in primary tumors and their paired nontumorous tissues in bulk of all 86 patients with ESCC. Staining intensity of *MT3‐MMP* in almost all nontumor tissues was scored greater than 4. Although, scores >4 were considered as expression of *MT3‐MMP* in normal esophageal tissues, down‐regulation of *MT3‐MMP* was defined as scores ≤4 (i.e., 0–4). In this context, down‐regulation of *MT3‐MMP* was found in tumor tissues, when compared to their paired nontumor tissues, in 66.3% (57/86) of ESCC patients. On the other hand, while *MT3‐MMP* expression in the horny pearl was seen in 41.2% (28/68) patients with moderately and well‐differentiated ESCC, *MT3‐MMP* positivity was detected in only 1 of 18 (5.5%) patients with poorly‐differentiated ESCC (*P *=* *0.004 compared to the former, Table**** 1). Further, down‐regulation of *MT3‐MMP* significantly correlated with frequency of either lymph node metastasis (*P *=* *0.039) or resected lymph node metastasis (*P *=* *0.017, Table**** 1). No correlation was observed between *MT3‐MMP* expression and age, gender, tumor invasion, or clinical stage (*P *>* *0.05, Table**** 1).

### *MT3‐MMP* down‐regulation is an independent factor for poor survival of ESCC patients

Moreover, Kaplan–Meier analysis was performed to analyze relation between *MT3‐MMP* expression and disease‐specific survival in 78 patients who were followed up till the end of the study. As shown in Figure**** 1D**, 5**‐year overall survival (OS) rate of ESCC patients with *MT3‐MMP* down‐regulation (score ≤4, median survival = 50.69 months) was significantly lower than those with score >4 (median survival = 30.77 months; *P *=* *0.029). Multivariate regression analysis shows that *MT3‐MMP* expression (Relative Risks = 0.389, *P *=* *0.020) and disease stage (Relative Risk = 10.276, *P *=* *0.001) were independent prognostic factors.

### *MT3‐MMP* inhibits growth of human ESCC cells by arresting at G1 phase

The correlation between *MT3‐MMP* down‐regulation and poor prognosis of ESCC patients raised a possibility that *MT3‐MMP* might negatively regulate progression of ESCC. To address this hypothesis, the human ESCC cell lines EC109 and EC9706 were stably transfected with *MT3‐MMP*‐expressing construct or empty vector as control, or conversely, infected with lentivirus encoding two different sequences of *MT3‐MMP* shRNA (designated as shMT3‐MMP‐1 or shMT3‐MMP‐2) or scrambled sequence as negative control. Stable clones with either overexpression or shRNA knockdown of *MT3‐MMP* were examined by Western blot analysis (see below in Fig. 3) and selected for further experiments.

**Figure 2:**
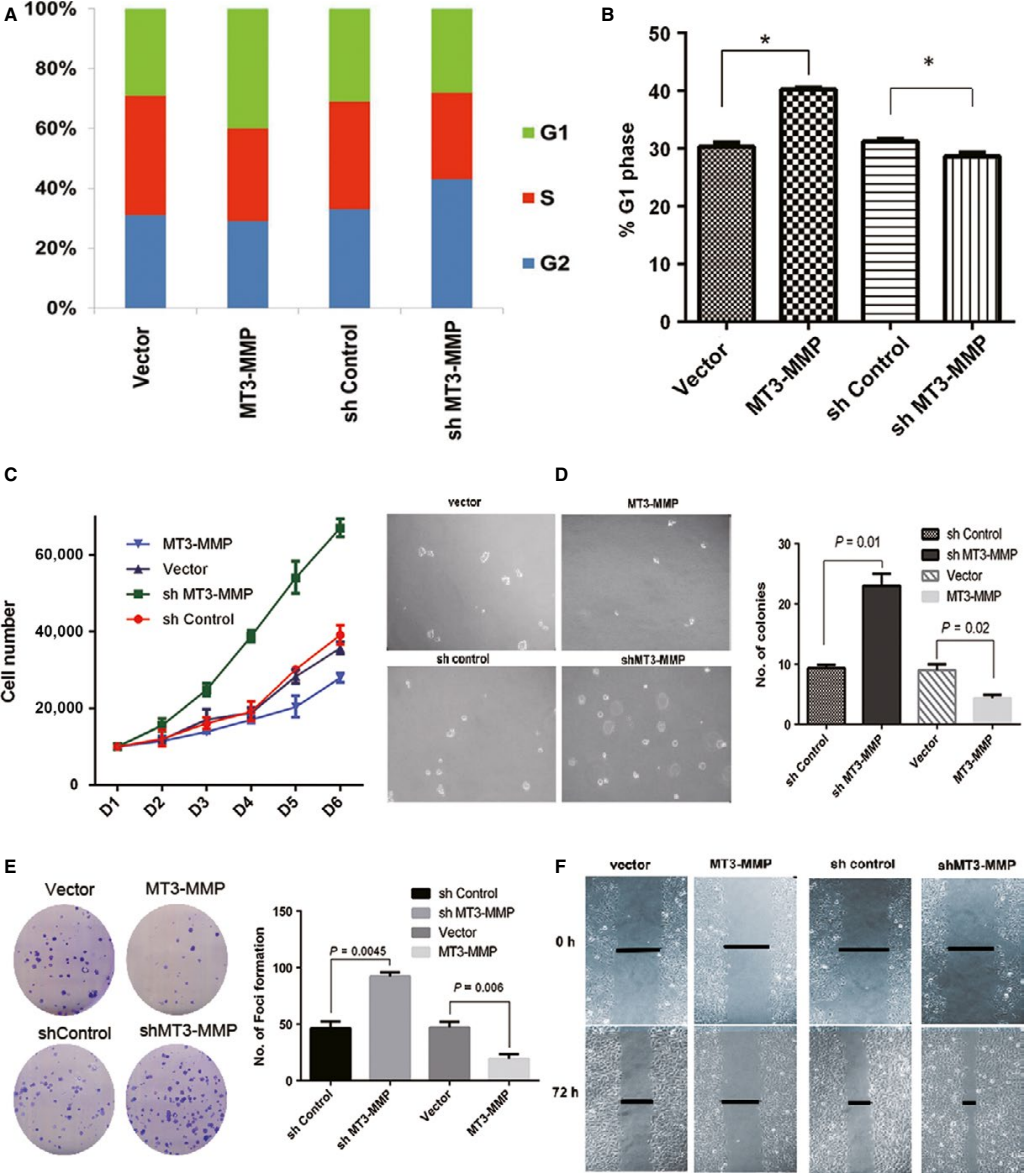
MT3‐MMP suppresses proliferation and migration of ESCC cells by arresting cell cycle at G1 phase. Human ESCC EC109 cells were stably transfected with a construct encoding human MT3‐MMP or empty vector, while EC9706 cells infected with lentivirus expressing shRNA specifically targeting human MT3‐MMP or a scrambled sequence as negative control. In vitro assays were then carried out using these cells. (A) Cells were stained with propidium iodide (PI), after which flow cytometry analysis was performed to monitor cell cycle distribution by determining DNA content. The percentages of cells in G1, S, and G2/M phases were shown. (B) Student's *t*‐test was performed to analyze differences between MT3‐MMP overexpression and empty vector, or shMT3‐MMP and shControl (*P <* *0.05). (C) Cell proliferation was assessed by counting the number of total cells for each condition. (D) Alternatively, the colony formation assay was conducted to monitor cell growth and division. The numbers of colonies were quantified for comparison between MT3‐MMT overexpression and empty vector, or shMT3‐MMP and shControl. (E) In parallel, the soft agar colony formation assay was carried out to determine anchorage‐independent cell growth, and colonies were counted for quantitative analysis. (F) Last, the wound healing assay was performed to examine cell migration. Representative images were shown for the intervals of 0 and 72 h after scratching. Lines indicate width of the scratches. The above error bars represent the mean ± SD of three independent experiments, *P < 0.05.

First, flow cytometry was performed to analyze effects of *MT3‐MMP* expression on cell cycle. As shown in Figure**** 2A and B, overexpression of *MT3‐MMP* significantly increased the percentage of EC109 cells ectopic in G1 phase (40.5% vs. 31.2% for shRNA control, *P *<* *0.05), while reduced S‐phase cells, at 24 h after release from synchronization by serum starvation. Conversely, shRNA knockdown of *MT3‐MMP* in EC9706 cells modestly but clearly decreased the percentage of G1 cells (28% vs. 30.9% for empty vector), while interestingly, it increased the number of cells in G2/M, indicting increased cell division (Fig.**** 2A). Moreover, shRNA knockdown of *MT3‐MMP* markedly increased proliferation of EC9706 cells, compared to shRNA control (*P *<* *0.05 for days 3–6, Fig**.** 2 **C**), while overexpression of *MT3‐MMP* moderately reduced cell growth (days 5–6).

### *MT3‐MMP* impairs colony‐forming and migration activity of human ESCC cells

Next, colony formation assays with or without soft agar were then carried out in human ESCC cells with either shRNA knockdown or ectopic overexpression of *MT3‐MMP*, as described above. Notably, depletion of *MT3‐MMP* dramatically increased the colony‐forming ability of EC9706 cells both on plate (*P *<* *0.05, Fig. 2D) and in soft agar (*P *<* *0.05, Fig.**** 2E). The latter indicated further that *MT3‐MMP* enhanced colony formation of ESCC cells independently of anchorage. In contrast, overexpression of *MT3‐MMP* clearly suppressed colony formation of EC109 cells (*P *<* *0.05 for either *MT3‐MMP* shRNA vs. shRNA control or *MT3‐MMP* overexpression vs. empty vector, Fig.**** 2E).

Last, as the clinical findings indicated that down‐regulation of *MT3‐MMP* was significantly associated with ESCC metastasis (Table**** 1), a wound healing assay was thus performed to assess the effect of MT3‐MMP on migration of human ESCC cells, reflecting metastatic potential of tumor cells in vitro. As shown in Figure**** 2F, although *MT3‐MMP*‐overexpressing EC109 cells displayed an impaired capability of cell migration, *MT3‐MMP* knockdown by shRNA dramatically promoted the speed of wound healing (representative images were shown in Fig. 2F; *P* < 0.05 for comparison of quantified data).

### *MT3‐MMP* up‐regulates the endogenous Cdk inhibitors p21^Cip1^ and p27^Kip1^ in ESCC cells

**Figure 3:**
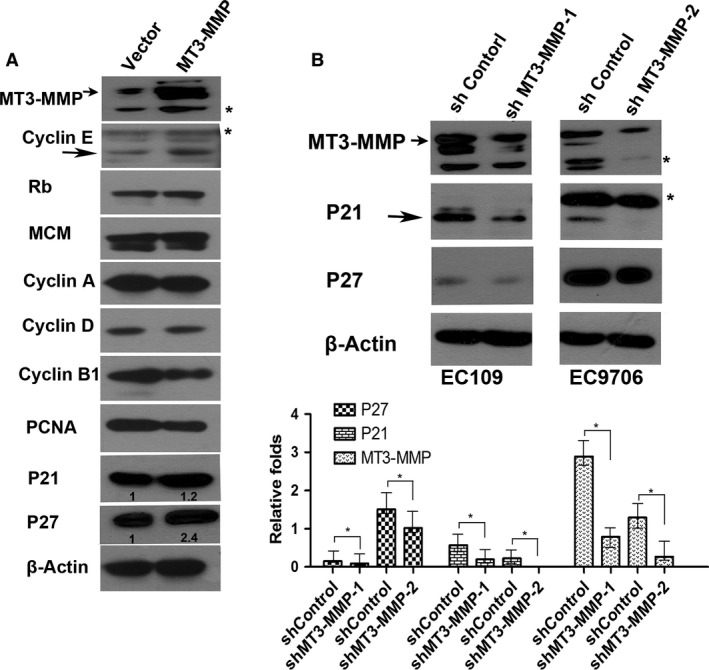
MT3‐MMP induces expression of the endogenous Cdk inhibitors p21^Cip1^ and p27^Kip1^. (A) EC109 cells stably expressing MT3‐MMP (top panel) were lysed and subjected to monitor expressions of cell cycle‐regulatory genes, including cyclins A, D, B1, E, MCM, Rb, PCNA, p21^Cip1^, p27^Kip1^. (B) EC109 and EC9706 cells were infected with lentivirus containing two shRNAs targeting different sequences of human MT3‐MMP (designated as shMT3‐MMP‐1 and shMT3‐MMP‐2, respectively) or their scrambled sequences as controls, after which Western blot analysis was performed to monitor protein levels of MT3‐MMP, as well as p21^Cip1^ and p27^Kip1^. Blots were quantified to determine relative expression (fold increase) of these genes by comparing to the housekeeping gene *β*‐actin. * indicates unknown band. Representative blots were shown. Similar results were obtained from two additional experiments.

To further investigate the potential mechanism(s) by which *MT3‐MMP* negatively regulates tumorigenesis and aggressiveness of ESCC, Western blot analysis was conducted to monitor expression of multiple key cell cycle‐regulatory proteins, including p21^Cip1^, p27^Kip1^, cyclin A, cyclin D, cyclin B1, cyclin E, MCM, Rb, and PCNA. As shown in Figure 3A, ectopic overexpression of *MT3‐MMP* in EC109 cells resulted in 1.2‐ and 2.4‐fold up‐regulation of p21^Cip1^ and p27^Kip1^, respectively, accompanied by a modest increase in cyclin E level, while no clear change was observed in protein levels of cyclin A, cyclin D, cyclin B1, cyclin E, MCM, Rb, and PCNA.

Conversely, shRNA knockdown of *MT3‐MMP* in either EC109 (shMT3‐MMP1,) or EC9706 (shMT3‐MMP2) substantially decreased about 70% and 90% in expression *MT3‐MMP*, respectively (Fig. 3B, upper panels). The shMT3‐MMP‐2 EC9706 cell line and its shRNA control were then used for the functional experiments as described above. Of note, knockdown of *MT3‐MMP* led to marked reductions in expression of p21^Cip1^ and p27^Kip1^ (Fig. 3B, lower panel). Together, these results indicate that *MT3‐MMP* negatively regulates proliferation and aggressiveness of ESCC cells by blocking progression of cell cycle from G1 to S and then G2/M phase (e.g., through the restriction point of cell cycle), likely in association with up‐regulation of the endogenous Cdk inhibitors p21^Cip1^ and p27^Kip1^. Importantly, they also provide further evidence supporting the notion that *MT3‐MMP* down‐regulation might contribute to tumorigenesis of ESCC, thereby correlating to poor prognosis of patients with ESCC.

## Discussion

MMPs belong to a family of zinc‐dependent endopeptidases. As proteolytic enzymes that degrade structural components of ECM, MMPs have originally been thought to promote cancer metastasis via disruption of basement membrane 6, 7. To this end, expression of MMPs is generally considered as an adverse factor for prognosis of cancer patients. However, with increasing number of MMPs as well as their functions that have been identified, the initial concept that MMPs positively correlate with cancer metastasis and thereby, poor outcome of patients has been challenged 16. In contrast to the tumorigenic activity of MMP2, MMP9, and *MT1‐MMP*, some MMPs (e.g., MMP8 and MMP11) might actually execute actions against tumor growth and metastasis 16 . For example, *MMP8* expression has been demonstrated as a favorable prognostic factor of patients with breast or oral cancer 16. We and Tatti, O et al. showed that overexpression of the membrane‐type matrix metalloproteinase *MMP16* is associated with poor clinical outcome and lymphatic invasion in gastric and melanoma cancer 19, 20. However, in this context, *MT3‐MMP*, a membrane‐anchored matrix metalloproteinase, joined this category in patients with ESCC. This study provides first evidence that in contrast to expression of *MT3‐MMP* in nontumor (normal) esophageal tissues, *MT3‐MMP* was down‐regulated at both mRNA and protein levels in primary ESCC tumor tissues, particularly those that were poorly differentiated. More importantly, *MT3‐MMP* down‐regulation in tumor tissues significantly correlated with higher rate of metastasis and poor survival of patients with ESCC. Therefore, these findings argue that similar to MMP8 in breast or oral cancer, *MT3‐MMP* might represent a favorable factor for prognosis of patients with ESCC.

Unlike *MT1‐MMP* and *MT2‐MMP* that contribute to aggressiveness and metastasis in most cancer types 21, expression of *MT3‐MMP* has been reported only in a few tumor types, including melanoma, gastric, pancreatic, and hepatocellular cancer 12, 13, 14, 15. In esophageal carcinoma, *MT1‐MMP* is highly expressed (e.g., the tumor/normal (T/N) ratio = 2.1), which is implicated in tumor aggressiveness and prognosis 22. Similarly, *MT2‐MMP* expression is found in 85.4% of primary tumors in esophageal cancer, but none or very weak in normal esophageal tissues, and positively correlates to angiogenesis and tumor size, while not to patients' survival 23. However, there is no report so far, to the best of our knowledge, regarding expression and role of *MT3‐MMP* in human ESCC. In this study, it was found that *MT3‐MMP* was down‐regulated in both 66.3% of primary ESCC tumors (by IHC, *n* = 86) and 63.3% of paired fresh surgically resected ESCC (by qPCR, *n* = 30), compared to nontumor esophageal tissues. Moreover, in sharp contrast to *MT1‐MMP* and *MT2‐MMP*,* MT3‐MMP* expression was significantly adversely correlated with lymph node metastasis and poor survival of patients with ESCC. In addition, although *MT3‐MMP* was expressed in moderately and well‐differentiated ESCC tissues, it was primarily localized within the horny pearl of tumor tissues.

Further in vitro experiments using human ESCC cancer cell line revealed that overexpression of *MT3‐MMP* inhibited tumor cell growth, whereas down‐regulation of *MT3‐MMP* by shRNA significantly promotes ESCC cell proliferation. In another sense, *MT3‐MMP* might play a role in inhibition of ESCC cancer cells. Moreover, down‐regulation of *MT3‐MMP* also enhanced colony‐forming and cell migration activity of ESCC cells, while overexpression of *MT3‐MMP* impaired these capabilities of tumor cells. Mechanistically, *MT3‐MMP* arrested ESCC cells at G1 phase by blocking G1/S transition, while down‐regulation of *MT3‐MMP* (e.g., by shRNA) drove ESCC cells entering G2/M phase, reflecting active cell division. These results were further supported by down‐regulation of p21^Cip1^ and p27^Kip1^, two key endogenous Cdk inhibitors that cease cell cycle progression by blocking activity of all Cdks, in ESCC cells with *MT3‐MMP* down‐regulation. While the exact mechanism(s) for regulation of p21^Cip1^ and p27^Kip1^ by *MT3‐MMP* remains to be defined, one possibility is that it may up‐regulate p21^Cip1^ and p27^Kip1^ via inactivation of MEK/ERK as MMPs belong to the ADAMTS family that antagonizes the EGFR/MEK/ERK signaling pathway 21. It is known that ERK activation leads to FOXM1 phosphorylation and nuclear translocation, which inhibits p21^Cip1^ expression 24, 25.

In summary, the present findings indicate that *MT3‐MMP* is down‐regulated in ESCC, which correlates to lymph node metastasis and poor survival of patients with this disease. They also suggest that *MT3‐MMP* might play a tumor‐suppressor role in progression of ESCC, probably through arresting tumor cells at G1 to prevent entry of cell cycle by down‐regulating p21^Cip1^ and p27^Kip1^. Considering *MT1‐MMP* is a primary driving force of tumor progression in most cancer types 26, including esophageal cancer 22; it has recently been reported that *MT3‐MMP* acts to antagonize *MT1‐MMP*‐driven tumor cell invasion 5. To this end, the present findings raise a possibility that down‐regulation of *MT3‐MM*P might release its brake on *MT1‐MMP* that is highly expressed in ESCC, thereby promoting tumor progression and aggressiveness. A better understanding of the tumor‐suppressive role of *MT3‐MMP* would significantly improve our knowledge in tumor progression of ESCC and prognosis of patients with this disease.

## Conflict of Interest

No potential conflicts of interest were disclosed.
